# Magnesium Implants: Prospects and Challenges

**DOI:** 10.3390/ma12010136

**Published:** 2019-01-03

**Authors:** Parama Chakraborty Banerjee, Saad Al-Saadi, Lokesh Choudhary, Shervin Eslami Harandi, Raman Singh

**Affiliations:** 1 Department of Chemical Engineering, Monash University, Clayton, VIC 3800, Australia; raman.singh@monash.edu; 2 Department of Mechanical and Aerospace Engineering, Monash University, Clayton, VIC 3800, Australia; saad.al-saadi@monash.edu (S.A.-S.); lokesh.gdu@gmail.com (L.C.); shervin.harandi@monash.edu (S.E.H.)

**Keywords:** magnesium alloy, implant, corrosion

## Abstract

Owing to their suitable mechanical property and biocompatibility as well as the technological possibility of controlling their high corrosion rates, magnesium and its alloys have attracted significant attention as temporary bio-implants. Though the ability of magnesium to harmlessly biodegrade and its inherent biocompatibility make magnesium alloys a suitable choice for a temporary implant, their high corrosion rates limit their practical application, as the implants can potentially corrode away even before the healing process has completed. Different approaches, such as alloying, surface modification, and conversion coatings, have been explored to improve the corrosion resistance of various magnesium alloys. However, the corrosion behavior of magnesium implants with and without a surface modification has been generally investigated under in-vitro conditions, and studies under in-vivo conditions are limited, which has contributed to the lack of translation of magnesium implants in practical applications. This paper comprehensively reviews the prospects of magnesium alloy implants and the current challenges due to their rapid degradation in a physiological environment. This paper also provides a comprehensive review of the corrosion mitigation measures for these temporary implants.

## 1. Introduction

The significant advancement in medical science and technology has considerably improved the quality and longevity of human life. The development of a variety of biomaterials is among the most notable innovations. Biomaterials are inorganic or organic materials that are designed to mimic physiological components and/or processes. Based on the material–host tissue interaction, biomaterials can be broadly classified into three categories [1]: (i) biotolerant materials; (ii) bioactive materials; and (iii) bioinert materials.

Implants are a type of biomaterials that are typically inserted into a host tissue to induce desired cellular behaviour and restore any impaired physical functions [2]. The application of implants varies depending on various requirements [3], such as (i) the healing and stabilization of the fractured bones by using screws, rods, and plates; (ii) the rectification of deformities, such as an abnormal spinal curvature; (iii) an improvement in the function of an organ and/or other parts of human body; and (iv) the replacement of a damaged and/or diseased part of the anatomy, such as damaged arthritic joints and malfunctioning heart valves.

The applicability of implants in a physiological environment mandates that they possess (i) excellent biocompatibility, requiring an adequate biological response; (ii) good mechanical properties; (iii) excellent corrosion resistance; and (iv) high resistance to fatigue [4,5]. Depending on their applications (such as orthopaedic, oral/dental, or cardiovascular), implants can be of different types. Table 1 shows the commonly used implants.

Among the various types of implant materials, metallic implants are widely used as orthopaedic, cardiovascular, and, in some cases, as oral implants. The traditional implant materials, including stainless steels, titanium alloys, and cobalt–chromium alloys, possess good resistance to corrosion, wear, and fatigue along with excellent load-bearing capabilities. However, it is well-known that these alloys also contribute to various negative effects, including stress shielding [6] and inflammation of local tissues due to a potential release of cytotoxic ions [7]. Additionally, when used as temporary implants (such as plates, screws, and wires) [8], a second surgery is generally required to remove the implant after the tissues have healed, which leads to increasing patient morbidity and healthcare costs [9,10]. Thus, implant materials that ‘biocorrode’, such as magnesium alloys, are currently attracting significant interest [11]. In this review, the required properties of temporary implants are discussed with a focus on magnesium-alloy-based temporary implants. The prospects and challenges of using magnesium-alloy-based implants are discussed in the following sections.

## 2. Temporary Implants

**Figure 1:**
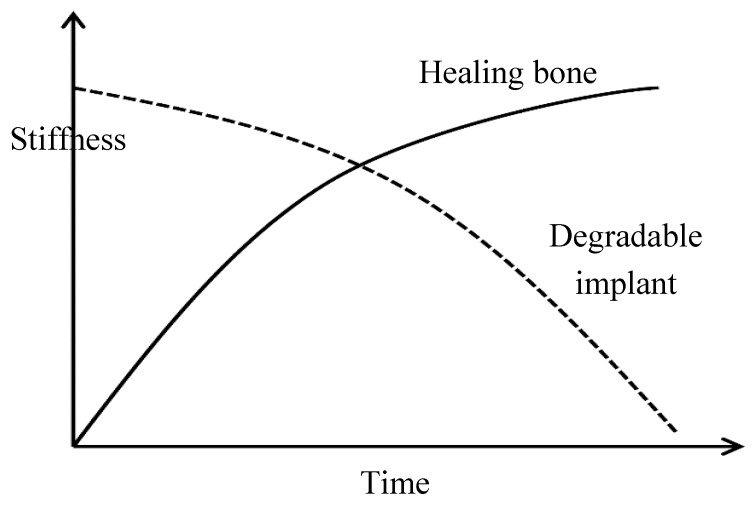
The gradual decrease in stiffness of a biodegradable magnesium implant and concurrent healing of the bone. Obtained from [13], with permission from Woodhead Publishing, 2018.

The low degradation rate of traditional implants has made them unsuitable for temporary implant applications, which has led to the rapid development of biodegradable implants. Polymers and metals are two material classes that have been widely considered for biodegradable temporary applications due to their inherent ability to dissolve away harmlessly within the human body, which eliminates the necessity of the removal of the implant after it has served its purpose [9,12]. In fact, during the healing process, the mechanical stiffness/strength of the temporary implants slowly decreases, while the surrounding tissue regains stiffness/strength (as shown in Figure 1). Harmless dissolution of the implant material eliminates the need for a second surgery that is commonly employed for removal of the implant once the tissue has completely healed [13].

Polymer-based temporary bi-implants are commercially available, and, in general, are made of either natural polymers (such as collagen, alginate, agarose, chitosan, fibrin) or synthetic polymers (such as poly-glycolic acid, poly-lactic acid, and poly-dioxanone) [14,15]. The principal advantage with polymer-based implants is that their microstructure and chemistry can be tailored easily to control their degradation rate in a physiological environment [15]. However, the mechanical properties of these polymers drastically decrease in a physiological environment, which limits their use as load-bearing implants. Typical degradation kinetics of a common poly-lactic acid-based implant in a chloride environment [16] are shown in Table 2. It is evident from Table 2 that the compressive strength and compressive modulus of the polymeric implants significantly decrease over time when immersed in a chloride environment. In this context, metal-based implants generally possess superior mechanical properties (Table 3), and are more suitable for most of the implant applications that involve load-bearing conditions. In fact, Wong et al. [17] have reported that the compressive strength of a magnesium-based metallic implant was 200 MPa after 4 weeks of immersion in simulated body fluid (SBF).

Magnesium and its alloys have attracted significant attention as a temporary implant due to their excellent biocompatibility with human physiology. In fact, magnesium is essential to the human metabolism as a cofactor for many enzymes [18,19], and magnesium ions (Mg^++^) are well-known to facilitate tissue-healing [20]. Additionally, extra Mg^++^ does not cause cellular toxicity in the human body and is generally expelled along with urine [21].

**Figure 2:**
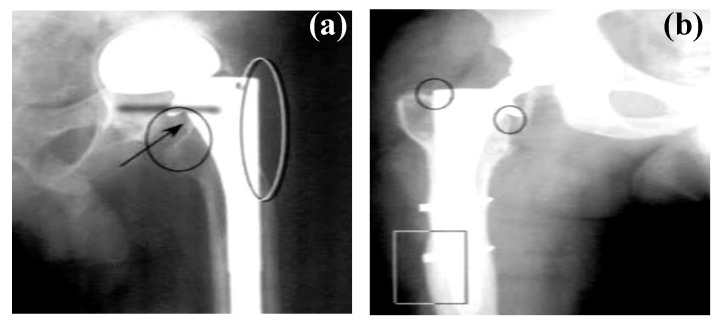
Radiographs of hip implants showing changes in bone mass due to stress shielding around an implant: (**a**) bone loss at the proximal region (indicated in the circled regions at the edge of the prosthesis) and (**b**) bone deposition (indicated by the rectangle) in the distal region of the implant . Obtained from [23], with permission from Wiley online library, 2018.

Even though good mechanical properties are essential for implants (especially orthopaedic implants), excessive stiffness of the traditional metallic implants can be detrimental to the healing bones or tissues as it can cause stress shielding [6]. Stress shielding occurs when a healing bone experiences stresses below a threshold as a result of the excessive stiffness of the implant material [6,22]. Figure 2a shows a radiograph of a femur in which significant bone loss was visible around the proximal end of the implant (indicated by the circled areas in Figure 2a) as a result of stress shielding [23]. Additionally, the reduction in the stress in the bone in the proximal region of the implant is sometimes accompanied by an increased stress in the distal regions. This results in deposition of additional bone due to increased stress transfer in the distal region (as is indicated by a rectangle in Figure 2b) [23].

In this context, the mechanical properties of magnesium and its alloys, i.e., low density (1.74–2.0 g/cm^3^) and elastic modulus (41–45 GPa), are more suitable for implant applications [9,21]. Table 3 presents a comparison of the mechanical properties of magnesium alloys, the traditional implant alloys, and natural bone [9,21]. It is evident from Table 3 that the density, elastic modulus, and yield strength of magnesium alloys are respectively the closest to those of natural bone. Therefore, magnesium alloys can significantly reduce the stress-shielding-related problems of orthopaedic/cardiovascular implants, and, most critically, in the situations of high-load-bearing implants.

In summary, magnesium implants offer several advantages, including (i) biocompatibility and osteogenesis [9,25,26,27,28], (ii) biodegradability and avoidance of a second surgery [18,19,20,21], (iii) favourable mechanical properties [9,21,24], (iv) machinability and dimensional stability [29], and (v) a high damping capacity [30].

## 3. Corrosion: The Major Drawback of Magnesium Implants

Despite their highly advantageous properties, magnesium and its alloys are not commonly used as degradable implants. The major drawback of magnesium alloys as implants is their exceptionally high corrosion rate in the physiological environment [19,31,32,33,34]. The high corrosion rates promote fast degradation of the mechanical properties, leading to the premature failure of implants before the completion of the tissue-healing process [34,35]. Additionally, hydrogen evolution, which is the principal cathodic reaction and concurrent with the corrosion of magnesium alloys [36,37,38,39,40], can significantly interfere with the healing process [10,41].

Human body fluid consists of water, electrolytic ions, proteins (such as albumins, globulins, and fibrinogen), and dissolved oxygen. Being the most electronegative engineering metal (with an electrochemical potential of −2.3 V versus a standard hydrogen electrode), magnesium is highly susceptible to corrosion in most aqueous environments, including human body fluid. Corrosion of magnesium produces an oxide/hydroxide layer on the surface, which is not protective in most aqueous environments. The following reactions represent the corrosion of magnesium alloys in most aqueous environments, including the physiological environment.

(1) $$\mathrm{Anodic}\;\mathrm{reaction}:\;Mg_{\left(s\right)}\to Mg_{\left(aq\right)}^{2+}+2e^{-}$$

(2) $$\mathrm{Cathodic}\;\mathrm{reaction}:\;2H_{2}O_{\left(aq\right)}+2e^{-}\to 2OH_{\left(aq\right)}^{-}+H_{2\;\left(g\right)}$$

(3) $$\mathrm{Overall}\;\mathrm{reaction}:\;Mg_{\left(s\right)}+2H_{2}O_{\left(aq\right)}\to Mg{\left(OH\right)}_{2\;\left(s\right)}+H_{2\;\left(g\right)}$$

Even though the formed hydroxide layer covers the surface of magnesium, this hydroxide layer is not stable in the presence of chloride ions in human body fluid. The presence of chloride ions quickly converts the hydroxide layer into highly soluble magnesium chloride [42].

(4) $$Mg{\left(OH\right)}_{2\;\left(s\right)}+2Cl_{\left(aq\right)}^{-}\to MgCl_{2}+2OH_{\left(aq\right)}^{-}$$

The disappearance of the hydroxide layer due to chloride ions hastens the corrosion of magnesium alloys. Additionally, hydrogen gas (H_2_) evolution during magnesium corrosion can create subcutaneous gas bubbles [43] and gas bubbles adjacent to the implants, which can cause the separation of tissues and/or tissue layers [36]. In-vitro studies report the critical tolerance level of hydrogen to be <0.01 mL/cm^2^/day, and this has been widely used to screen magnesium alloys for temporary implant applications [36]. However, it should be noted that each alloy must be investigated in-vivo in relation to the intended function. In this context, it may be interesting to note that a few in-vivo studies [28,44] have reported the evolved hydrogen-forming gas pockets only in the first week post-surgery, which gradually disappear over a period of 2–3 weeks post-surgery. Thus, it may be reasonable to assume that hydrogen evolution may not significantly interfere with the healing process as long as the corrosion rates of the magnesium implants are appropriately controlled in the first couple of weeks, such that the hydrogen evolution rate remains lower than 0.01 mL/cm^2^/day.

In order to identify ways to improve the corrosion resistance of magnesium implants, it is important to investigate their corrosion rates and mechanisms in the presence of a physiological environment. In-vitro corrosion of magnesium alloys has been widely investigated in a variety of simulated physiological solutions, which is summarized in the following sections.

## 4. In-Vitro Corrosion of Magnesium Implants in Various Simulated Physiological Environments

Corrosion of magnesium alloys significantly depends on the ions present in the electrolyte. Magnesium alloy corrosion has been investigated in a variety of in-vitro electrolytes that mimic the composition of the physiological fluid. Depending on the various ion concentrations in these electrolytes, different corrosion rates and mechanisms have been observed for a variety of magnesium implants [45,46,47,48]. The most commonly employed in-vitro electrolytes are Hank’s solution, Ringer’s solution, Dulbecco’s Modified Eagle’s Medium (DMEM), phosphate-buffered saline (PBS), original simulated body fluid, conventional simulated body fluid (c-SBF), ionized simulated body fluid (i-SBF), revised simulated body fluid (r-SBF), and modified simulated body fluid (m-SBF) [45,46,47]. It is evident from Table 4 that the composition and the ion concentrations significantly vary among these in-vitro electrolytes. It is to be noted here that a variety of buffers, including HEPES, Tris-HCl, and HCO_3_^−^, are used in these in-vitro electrolytes, which can consume the OH^−^ ions generated during magnesium corrosion, and, thereby, significantly alter the true corrosion rate of magnesium implants in in-vitro systems [48].

Among the in-vitro electrolytes, various SBFs have been extensively used due to their close similarities in composition with blood plasma (i.e., the inorganic ion concentration as shown in Table 4). Additionally, SBF can promote bone-like apatite formation on a variety of substrates [49,50,51]. Among other in-vitro electrolytes, Hank’s balanced salt solution (HBSS) is one of the most prominent ones. HBSS has high nutritional value [52] and is generally used in biomedical research to support a variety of cell cultures [19]. One of the major differences between SBF and HBSS is the presence of a buffering additive in the case of SBF. In general, the buffering additives can form complexes with calcium ions (Ca^2+^) [53,54], which interferes with the corrosion process of a metallic implant [55].

### 4.1. Influence of Inorganic Ions Present in the In-Vitro Electrolytes

The nature of the inorganic ions present in the in-vitro electrolytes (Table 4) can significantly affect the corrosion rate of magnesium implants. In general, a hydroxide layer (Mg(OH)_2_) that forms on a magnesium alloy’s surface is rapidly dissolved in the presence of chloride ions (Cl^−^) [57,58]. The high Cl^−^ concentrations in blood plasma as well as in the in-vitro electrolytes explain the rapid corrosion of magnesium implants. Sulphate ions (SO_4_^2−^) are also well-known to accelerate the corrosion of magnesium implants. On the other hand, bicarbonate ions (HCO_3_^−^) in appreciable quantities (>27 mmol L^−1^) can form an insoluble carbonate, which can passivate the magnesium’s surface [48]. Low concentrations of phosphate ions (HPO_4_^2−^) can also decelerate magnesium corrosion by the formation of insoluble and dense phosphates. In addition to these anions, inorganic cations can also affect the corrosion of magnesium in the in-vitro electrolytes [36]. Song et al. [36] reported that the presence of calcium ions and phosphate ions can lead to the formation of calcium phosphate, which, when precipitated on the magnesium’s surface, can significantly retard the corrosion kinetics.

### 4.2. Influence of Organic Components Present in the In-Vitro Electrolytes

Even though the electrolytes in most of the in-vitro studies did not contain organic components, the actual blood plasma does contain them in appreciable quantities. Hence, it is important to understand the influence of the most prominent organic components present in blood plasma on the corrosion resistance of magnesium alloys. Proteins, glucose, fatty acids, and cholesterol are the most common organic components in blood plasma. Proteins are well-known to adsorb on a metal’s surface and can retard the corrosion of a metallic implant [59], as also reported by Yamamoto et al. [60]. They attributed this to the adsorption of protein on the metallic implant surfaces, which acts as a corrosion barrier, and significantly reduces the corrosion rate of magnesium implants. However, Harandi et al. [22] reported that protein (bovine serum albumin) adsorbs on the implant’s surface during the initial hours of immersion, but, with increasing time of immersion in the Hank’s solution, the protein chelates with the corrosion products, causing a disruption of the protective film and, thus, accelerating the corrosion of the metallic implant.

## 5. Protective Coatings to Improve the Corrosion Resistance of Magnesium Implants

Various strategies, including alloying [61], surface modification using energetic radiation [62,63], and conversion coatings [64], have been employed to improve the corrosion resistance of magnesium alloys. Among these, the application of conversion coatings is the most widely used strategy. Biocompatibility and good barrier properties are the key criteria that a coating must satisfy in order for the coated magnesium alloys to be used as implants. In the following section, different conversion coatings that have been developed to improve the corrosion resistance of magnesium implants are summarized.

### 5.1. Biodegradable Polymeric Coatings

**Figure 3:**
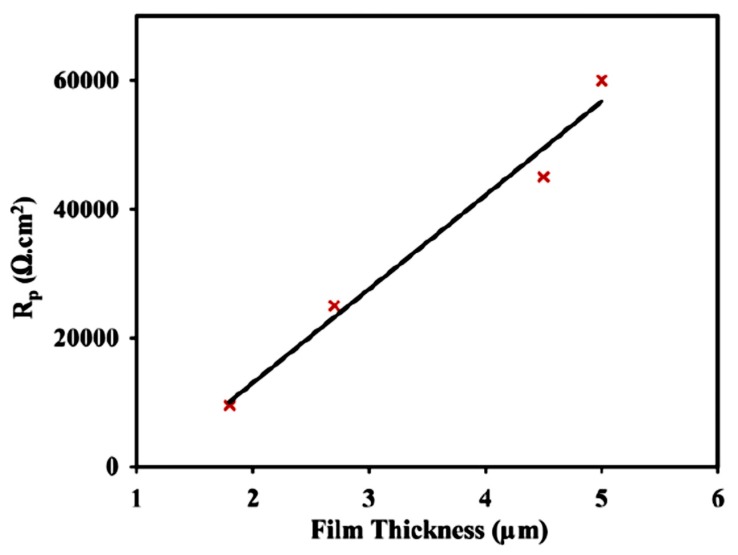
The increase in corrosion resistance of the poly-lactic acid (PLA)-coated AZ21 alloy with increasing coating thickness. Obtained from [65], with permission from Thin Solid Films, Elsevier, 2018.

A variety of biodegradable polymers, including poly-lactic acid (PLA) [65], poly(lactic–co-glycolic acid) [66], and polycaprolactone (PCL) [67], have been used to improve the corrosion resistance of magnesium implants. Alabbasi et al. [65] spin-coated polylactic acid (PLA) onto AZ21 magnesium alloy and reported that this coating significantly improved the corrosion resistance of this alloy in simulated body fluid. The corrosion resistance (Rp, as shown in Figure 3) increased while the adhesion of the coating decreased with the increasing coating thickness.

**Figure 4:**
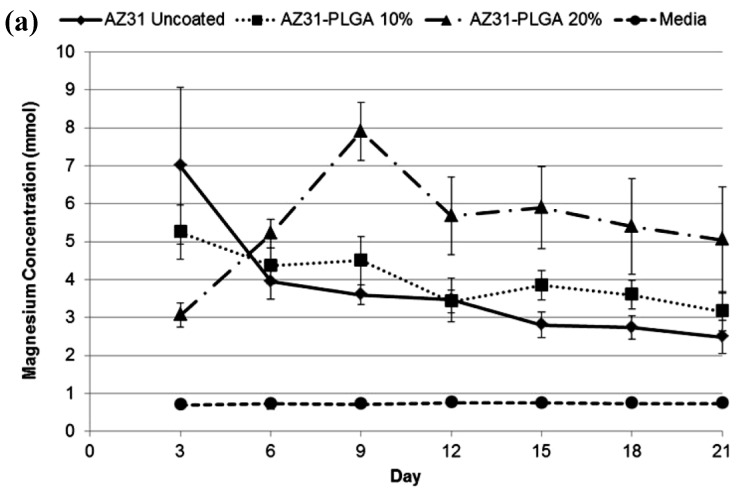

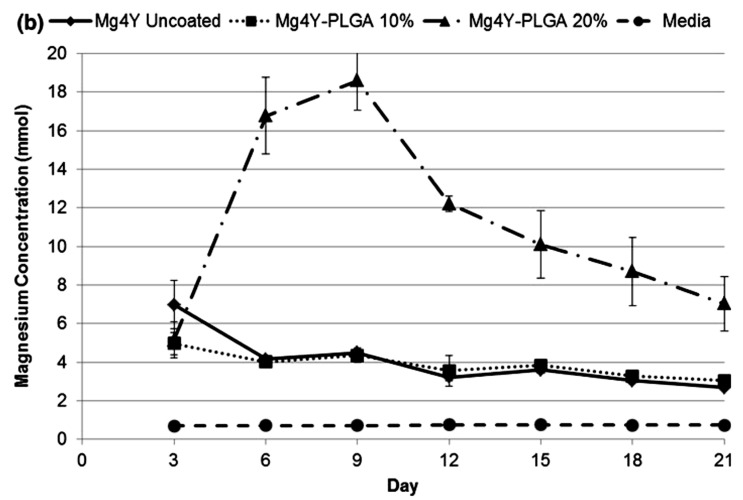
The magnesium ion concentration of the poly (lactic-co-glycolic acid) polymer-coated (**a**) AZ21 and (**b**) Mg4Y alloy substrates in DMEM at various days of immersion. Obtained from [65], with permission from Thin Solid Films, Elsevier, 2018.

Ostrowski et al. [66] investigated a poly(lactic-co-glycolic acid) polymer coating on AZ31 and Mg4Y magnesium alloys. The application of the poly(lactic-co-glycolic acid) polymer film resulted in a lower I_corr_ and a less negative E_corr_ of the polymer-coated Mg alloys pre-exposed to Dulbecco’s modified Eagle’s medium (DMEM). Although the concentrations of the magnesium ions reduced during the first 3 days of immersion of the polymer-coated AZ31 and Mg4Y alloys in DMEM, the ion concentrations increased with a further increase in the duration of immersion (Figure 4). As shown in Figure 4 [66], both uncoated and coated AZ31 respectively possess a higher resistance to corrosion compared to the Mg4Y alloy.

**Figure 5:**
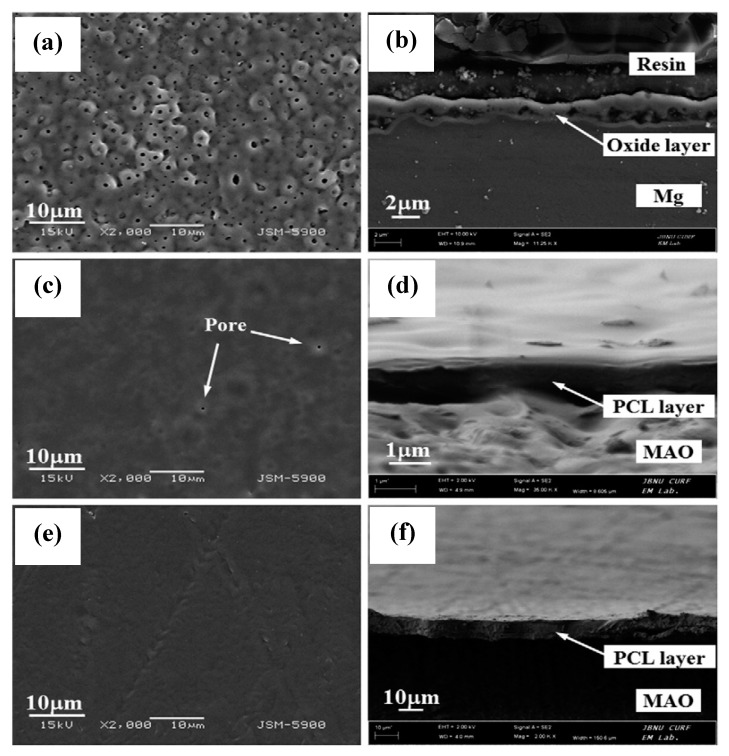
Surface morphology (**a**,**c**,**e**) and cross-section images (**b**,**d**,**f**) of the (**a**,**b**) micro-arc oxidation (MAO)-treated, (**c**,**d**) MAO-4% PCL duplex-coated, and (**e**,**f**) MAO-7% PCL duplex-coated magnesium. Obtained from [67], with permission from Thin Solid Films, Elsevier, 2018.

Shi et al. [68] reported that the adhesion of PLA coatings on the magnesium alloy can be significantly improved by a surface pre-treatment using micro-arc oxidation (MAO). The adhesion strength test of a PLA/MAO coating on the AZ31 magnesium alloy showed that MAO improved the bonding of the PLA film to the MgO layer. The PLA/MAO coating significantly improved the corrosion resistance of AZ31 alloys. However, Li et al. [67] reported that MAO pre-treatment can potentially facilitate the development of a porous coating with cracks. Thus, they [67] developed a duplex coating composed of a MAO (an alkaline silicate-flouride-based electrolyte) inner layer and a polycaprolactone (PCL) top biodegradable layer. SEM images revealed the formation of micropores (~1 µm) on the just MAO-treated surface (Figure 5a). However, the coating prepared with 4 wt% of PCL produced a uniform polymer layer with much fewer micropores (0.5 µm) over the crater-like MAO surface (Figure 5c). Increasing the PCL concentration to 7% formed a uniform and pore-free coating with complete coverage over the MOA-coated Mg (Figure 5e). The cross-sectional view showed that the thickness of the coating developed with 7% PCL was 3 times higher than that of the MOA treatment. Potentiodynamic polarization test results indicated that the coating developed with 7% PCL provided the best corrosion resistance in Hanks’ solution. Tian et al. [69] also reported that a duplex coating, composed of PCL and plasma electrolytic oxidization PEO layers, significantly improved the corrosion resistance of an AZ31 magnesium alloy. A further modification of the PEO/PCL coating with polydopamine (PDAM) showed no obvious improvement in corrosion resistance, resistance to hydrogen evolution, and cytocompatibility. However, the surface became more suitable for cell adhesion after the PDAM treatment [69].

Hanas et al. [70] deposited a PCL–nanofibrous layer on an AZ31 magnesium alloy using an electrospinning technique. Randomly aligned and uniform nanofibers with an average diameter of 400 nm were deposited on the alloy’s surface due to the PCL coating. They concluded that the PCL coating reduced the rate of increase of pH and the rate of H_2_ evolution, facilitated the deposition of Ca–P, improved the biomineralization, and inhibited pitting corrosion during immersion in supersaturated simulated body fluid. Xi et al. [71] synthesized a composite coating containing a hyperbranched polyphenylene sulphide (HPPS), polycaprolactone (PCL), and zinc oxide (ZnO) with a view to decreasing the corrosion rate of AZ91 alloy in SBF [71]. While the PCL coatings formed an interconnected pore network, the addition of ZnO and HPPS significantly reduced the porosity and improved the uniformity of the resultant coating. The HPPS/PCL/ZnO composite coating possessed superior corrosion resistance as compared to PCL and PCL/ZnO coatings. In another study, polycaprolactone (PCL) and zinc-oxide nanoparticle (ZnO NP) composite coatings were deposited on AZ31 by electrospinning, which also improved the corrosion resistance of this alloy in simulated body fluid [72]. Surface pre-treatments, such as micro-arc oxidation and plasma electrolytic oxidation, and the inclusion of metal particles, such as ZnO and TiO, seem to improve the uniformity and adhesion and reduce the porosity of the polymeric coatings, which results in a significant improvement in the corrosion resistance of magnesium implants.

### 5.2. Biodegradable Silane Coatings

Silane coatings have been widely used to improve the corrosion resistance of various magnesium alloys in different corrosive environments. A few different types of silanes have been considered as biocompatible [73] and have been reported to improve the corrosion resistance of a few magnesium implants. Jiang et al. [74] have reported that an amine silane treatment on a bioglass fibre had little negative effect on the biological response of these fibres. Muller et al. [75] reported that a collagen and amino silane coating synthesized on stainless steel facilitated cell proliferation and adhesion to the substrate. Additionally, Glycidoxypropyltrimethoxy silane has been routinely used as a bioactive and biocompatible binder and precursor for the synthesis of porous gelatine–siloxane hybrids for bone tissues [76,77].

**Figure 6:**
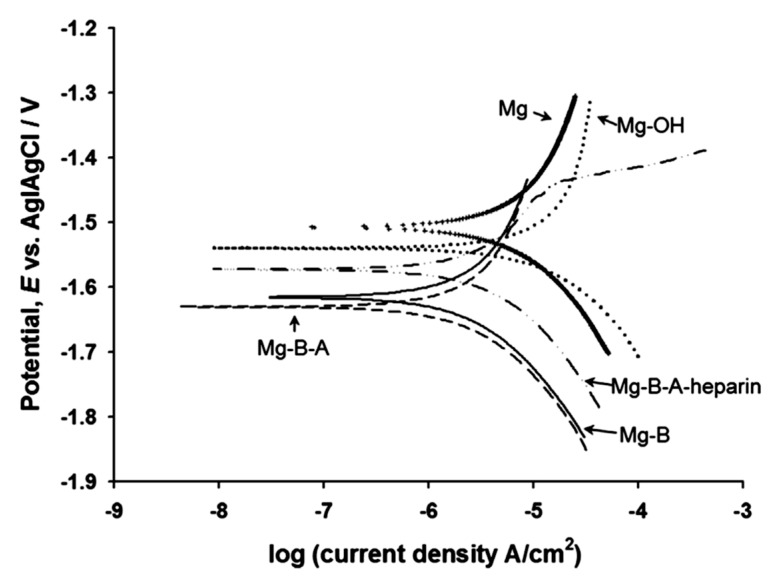
The potentiodynamic polarization of the uncoated, hydroxide pre-treated, BTSE-coated, γ-APS-coated, BTSE/γ-APS-coated, and BTSE/γ-APS/heparin-coated AZ31 alloy in simulated body fluid. Obtained from [78], with permission from Acta Biomaterialia, Elsevier, 2018.

Liu et al. [78] used a two-step duplex coating on a hydroxide pre-treated AZ31 magnesium alloy. In the first step, they synthesized a bistriethoxysilylethane (BTSE) coating, and, in the second step, they synthesized a 3-aminopropyltrimethoxysialne (γ-APS) coating on the BTSE-coated AZ31 substrate. To further improve the bioactivity of the BTSE/γ-APS-coated AZ31 alloy’s surface, they attached heparin on the coated surface. They reported that, even though the BTSE/γ-APS coating significantly improved the corrosion resistance of the AZ31 substrate, the inclusion of heparin deteriorated the corrosion resistance of the coated implant (Figure 6).

Gaur et al. [79] synthesized a series of silane mixture coatings using different ratios of diethylphosphateoethyltriethoxysilane (DEPETES) and bis-[3-(triethoxysilyl) propyl]tetrasulfide (BTESPT). They reported that a coating developed by mixing DEPETES and BTESPT in a ratio of 1:4 significantly improved the corrosion resistance of a Mg-6Zn-Ca alloy and delayed the corrosion of this alloy for the entire duration of immersion (175 h) in modified simulated body fluid. In a subsequent study, Gaur et al. [80] reported that glycidoxypropyl–trimethoxysilane, when mixed with methyltriethoxy-silane in a ratio of 3:1, significantly improved the corrosion resistance of a Mg-6Zn-Ca alloy in modified simulated body fluid.

Cordoba et al. [81] synthesized a glycidoxypropyl–trimethoxysilane coating modified with titanium iso-propoxide that significantly improved the corrosion resistance of AZ31 alloy during immersion in simulated body fluid (SBF) for 7 weeks. However, when applied to ZE41 alloy, this coating showed limited corrosion protection after 7 weeks of immersion in SBF. Al-Saadi et al. [82] impregnated glycidoxypropyl–trimethoxysilane with hexagonal boron nitride and applied this coating to WZ21 alloy. They reported that this biocompatible coating significantly improved the corrosion resistance of WZ21 alloy in simulated body fluid. Most of the reports [78,79,80,81] related to silane coatings on magnesium implants concluded that the hydrophobic nature of silane inhibited the ingress of corrosive ions to the metal substrate’s surface, which explains the significantly improved corrosion resistance of magnesium implants due to the silane coatings.

### 5.3. Biodegradable Calcium Phosphate Coatings

**Figure 7:**
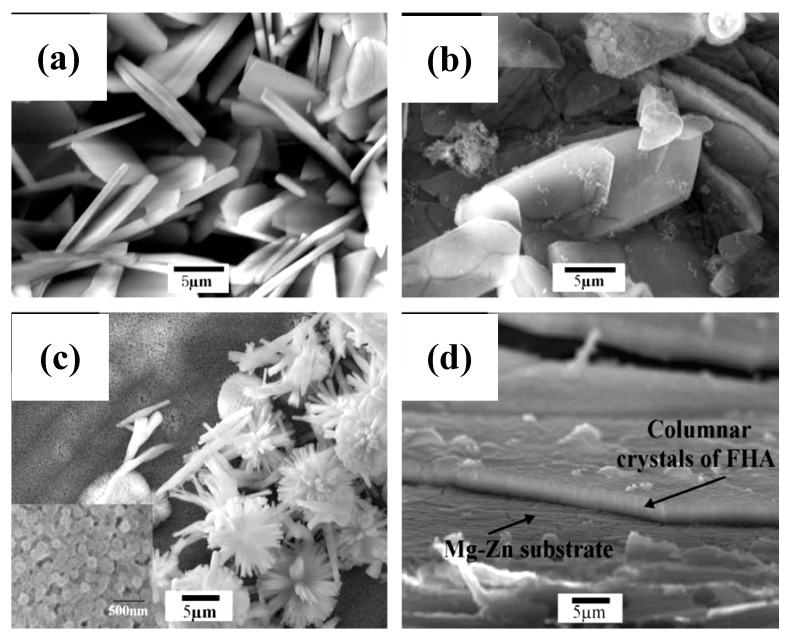
Surface morphologies of: (**a**) brushite-coated, (**b**) hydroxyapatite-coated, and (**c**) fluoridated-hydroxyapatite-coated Mg–Zn alloy substrates. (**d**) Cross-sectional view of the fluoridated-hydroxyapatite-coated specimen. Obtained from [84], with permission from Acta Biomaterialia, Elsevier, 2018.

Calcium (Ca) and phosphorous (P) are the two major elements present in bone minerals [83,84]. Thus, the surface modification of magnesium alloys with Ca–P coatings can potentially improve the biocompatibility of, and facilitate osteointegration around, the coated implants [84]. Song et al. [84] electrodeposited three different types of Ca–P coatings, brushite (CaHPO_4_.2H_2_O), hydroxyapatite (Ca_10_(PO_4_)_6_(OH)_2_), and fluoridated hydroxyapatite (Ca_5_(PO_4_)_3_(OH)_1−x_F_x_), on a Mg–Zn alloy. The morphologies of the three coatings and the cross-section of the fluoridated hydroxyapatite-coated substrate are shown in Figure 7. Even though all three coatings improved the corrosion resistance of the magnesium alloy, the brushite-coated specimen showed delayed bone-like apatite formation as compared to the hydroxyapatite- and fluoridated-hydroxyapatite-coated specimens, both of which promoted the nucleation of osteoconductive minerals (apatite or β-TCP) for one month. Since the hydroxyapatite coating synthesized through the alkali heat treatment of brushite was fragile and less stable, they concluded that the fluoridated hydroxyapatite coating was superior for improving the bioactivity and corrosion resistance of the Mg–Zn alloy substrates.

**Figure 8:**
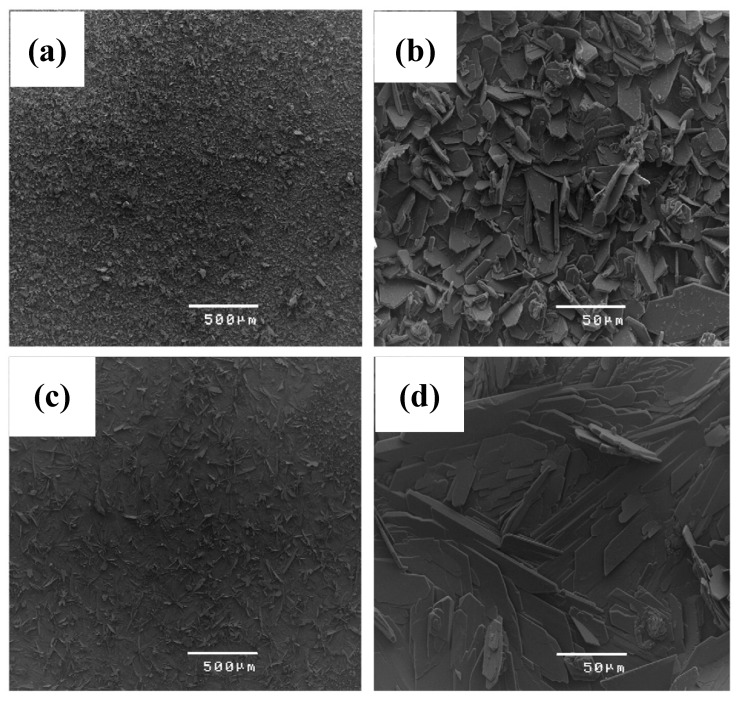
Surface morphologies of the Ca–P coating synthesized using (**a**,**c**) constant potential and (**b**,**d**) pulse potential techniques. Obtained from [87], with permission from Materials Letters, Elsevier, 2018.

Liu et al. [85] used a hydrothermal method to synthesize nanostructured dibasic calcium phosphate, which significantly improved the corrosion resistance of an AZ31B magnesium alloy. Jafari et al. [86] has also reported improvement in the corrosion resistance of an AZ91D alloy due to a Ca–P coating. Kannan et al. [87] employed an innovative pulse-potential technique to synthesize a Ca–P coating on an AZ91D alloy. There was a distinct morphological difference between the Ca–P coating developed using a constant potential and the pulse potential (Figure 8). The coating synthesized using the pulse potential showed higher corrosion resistance as compared to that synthesized using a constant potential. Wang et al. [88] had also used a pulsed electrodeposition technique to synthesize a calcium-deficient hydroxyapatite coating on a Mg-Zn-Ca alloy, which provided an order of magnitude improvement in corrosion resistance to the uncoated alloy. Meng et al. [89] used pulse electrodeposition to synthesize a fluorine-doped hydroxyapatite coating, which significantly improved the corrosion resistance of a Mg-Zn-Ca alloy. Additionally, they had reported that the nanophase and the high surface area of the fluorine-doped hydroxyapatite coating facilitated the precipitation of Mg^2+^, Ca^2+^, and PO_4_^3−^ ions.

**Figure 9:**
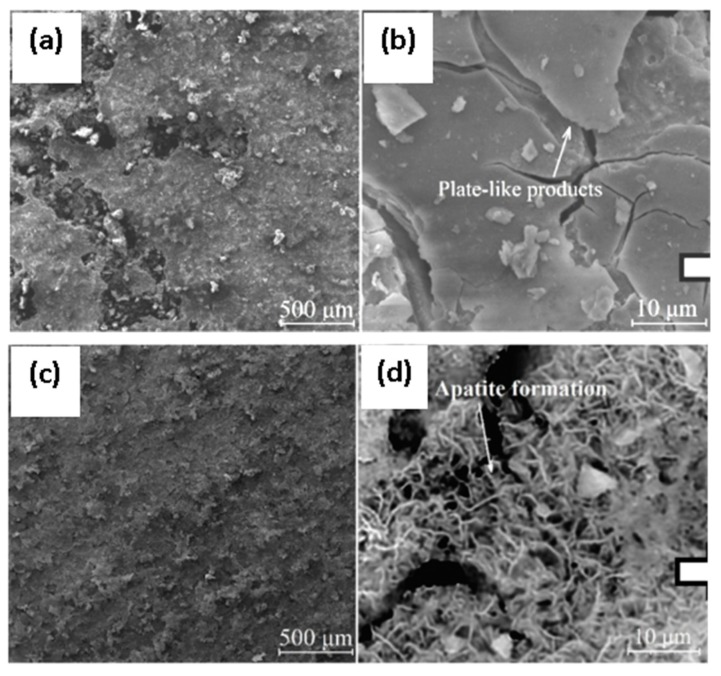
Post-corrosion morphologies of the (**a**,**b**) uncoated and (**c**,**d**) nanostructured-hydroxyapatite-coated alloy substrate after 7 days of immersion in simulated body fluid. Obtained from [91], with permission from Materials Science and engineering C, Elsevier, 2018.

Surmeneva et al. [90] used radio frequency magnetron scattering to deposit a thin film (550–750 nm thick) of hydroxyapatite on a Mg–Ca alloy. Potentiodynamic polarization tests suggested that this coating was able to suppress the cathodic reactions; however, the anodic current densities of the coated alloy were similar to that of the uncoated one. A radio frequency magnetron technique was also used by Mukhametkaliyev et al. [91] to synthesize a dense nanostructured hydroxyapatite coating with low porosity on an AZ91 alloy. This nanostructured coating significantly improved the corrosion resistance of the alloy as well as improved its bioactivity via apatite formation. The post-corrosion morphologies of the uncoated and coated specimens are shown in Figure 9.

Ren et al. [92] have reported an innovative microwave-assisted technique to synthesize a dense and uniform calcium-deficient hydroxyapatite coating on an AZ31 alloy. The coating significantly improved the corrosion resistance of the alloy in simulated body fluid and promoted the formation of a dense apatite layer after 7 days of immersion. Additionally, a cell culture study revealed that this coating stimulated cell proliferation during 5 days of incubation. Abdal-hay et al. [93] synthesized a composite coating using nanostructured hydroxyapatite and poly(ε-caprolactone). The poly(ε-caprolactone) coating with and without nanostructured hydroxyapatite improved the corrosion resistance; however, the poly(ε-caprolactone) coating with nanostructured hydroxyapatite promoted the formation and uniform distribution of apatite particles. Subsequently, Abdal-hay et al. [94] synthesized a hydroxyapatite-doped poly(lactic acid) coating on an AZ31B alloy. They reported that the addition of hydroxyapatite improved the hydrophobicity of the coating. This coating also improved the corrosion resistance and promoted apatite formation on the AZ31B alloy. Zomorodian et al. [95] synthesized a composite coating using polycaprolactone, nanostructured hydroxyapatite, and ceplalexin. They concluded that, even though the polycaprolactone coating by itself provides corrosion protection to AZ31 alloy in Hanks’ solution, the addition of ceplalexin and nanostructured hydroxyapatite to the polycaprolactone coating decreased the corrosion protection offered by the coating.

### 5.4. Graphene-Derivative-Based Coatings

**Figure 10:**
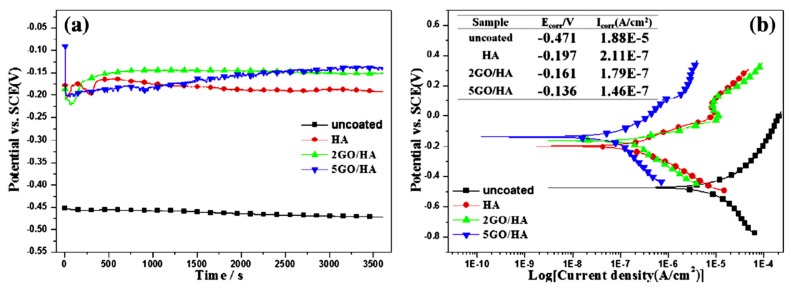
(**a**) open circuit voltage versus time and (**b**) potentiodynamic polarization plots of the uncoated, hydroxyapatite-coated, and graphene oxide (GO)/hydroxyapatite-coated titanium substrate. Obtained from [101], with permission from Carbon, Elsevier, 2018.

Recently, graphene and graphene derivatives have attracted significant attention as coatings for implants. These coatings have been reported to facilitate adhesion and the proliferation of the human-osteoblast-like cell line, and show potential to facilitate the differentiation of mesenchymal stromal cells into the osteoblast lineage [96,97,98]. Kim et al. [99] converted free-standing graphene/calcium carbonate (CaCO_3_) films into graphene/hydroxyapatite composites after incubating them in simulated body fluid for 4 days. Lahiri et al. [100] have reported that low concentrations of graphene fillers can improve the biocompatibility to bone cells. In one of the first reports, Li et al. [101] reported a higher open-circuit potential (Figure 10a) and lower anodic and cathodic current densities (Figure 10b) due to graphene oxide/hydroxyapatite composite coatings, and found a significant improvement in the corrosion resistance of a titanium substrate due to these coatings. This concept was subsequently extended to magnesium implants.

**Figure 11:**
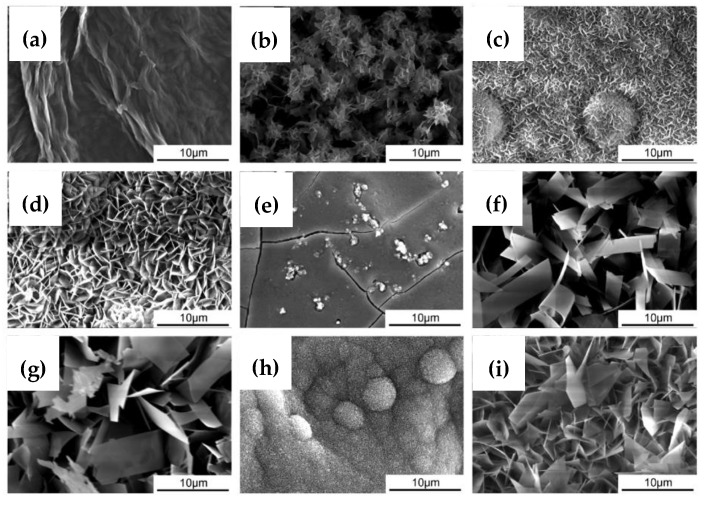
Surface morphologies of (**a**) the graphene oxide coating on AZ91. (**b**–**d**) the graphene oxide/hydroxyapatite coating with a growth time of 6 h, 1 day, and 2 days, respectively, at a pH of 6.65, (**e**–**g**) the hydroxyapatite-only coating with a growth time of 6 h, 1 day, and 2 days, respectively, at a pH of 6.65. A graphene oxide/hydroxyapatite coating grown for 2 days at a pH of (**h**) 6.4 and (**i**) 6.9. Obtained from [102], with permission from Materials Letters, Elsevier, 2018.

**Figure 12:**
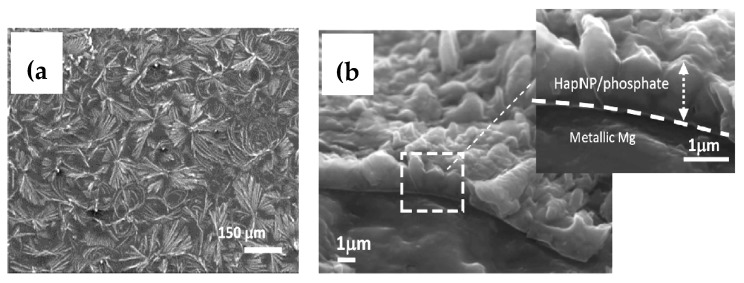
The (**a**) surface morphology and (**b**) cross section of a graphene oxide/hydroxyapatite nanoparticle coating on a high-purity magnesium substrate. Obtained from [103], with permission from Applied Surface Science, Elsevier, 2018.

Gao et al. [102] synthesized a 34-µm thick graphene oxide/hydroxyapatite coating on an AZ91 alloy. The alloy substrate was, at first, dip-coated by graphene oxide (GO), and was then exposed to simulated body fluid, which facilitated the biomimetic mineralization of hydroxyapatite on the GO coating. They reported that graphene oxide promoted the nucleation and subsequent growth of a dense and uniform hydroxyapatite layer. Figure 11 shows the surface morphologies of graphene oxide/hydroxyapatite coatings as a function of solution pH and duration of immersion in modified simulated body fluid. These coatings were reported to significantly improve the corrosion resistance of the magnesium alloy substrates in simulated body fluid. Santos et al. [103] synthesized an ultrathin (1 µm) graphene oxide/hydroxyapatite nanoparticle coating on an ultra-high purity magnesium substrate. The surface morphology and the cross-section of this coating are shown in Figure 12. However, the coating provided little corrosion protection to the magnesium alloy substrate.

## 6. Conclusions

The ability of biocompatible magnesium and its alloys to easily corrode in human body fluid without generating any toxic products makes them one of the most suitable candidate materials for biodegradable temporary implants. However, the very high corrosion rate limits their use as an implant as it may potentially degrade even before the healing has been completed. There are a few different approaches to improve the corrosion resistance of magnesium implants. Various strategies, including alloying, surface modification using energetic radiation, and conversion coatings, have been employed to improve the corrosion resistance of various magnesium alloys. Among these strategies, conversion coatings have been the most widely used approach for a variety of magnesium alloys. Biocompatibility and good barrier properties are the key criteria that a coating must satisfy in order to be used on implants. Various different types of coatings, including polymeric coatings, calcium–phosphate coatings, and graphene coatings have significantly improved the corrosion resistance of magnesium and its alloys. However, one of the major drawbacks in the reports on the corrosion behavior and coatings of magnesium implants has been the lack of data under in-vivo conditions. Even though there has been a consensus in the literature about the major differences in in-vivo and in-vitro conditions, little has been reported on the corrosion behavior of the implants and/or the performance of the coating synthesized on the implants. The prospects of magnesium alloys as temporary implants will significantly depend on the in-vivo performance evaluation of these various biodegradable coating systems.

## Figures and Tables

**Table 1:** Classification of different types of traditional implant materials.

Types of Implants	Materials Used
Metal	Titanium alloys, stainless steel, gold alloys, cobalt–Chromium alloys, platinum electrodes
Polymers	Poly(methyl-methacrilate), Polyethylenes, polypropelenes, polyamides, polyesters, hydrogels
Ceramics	Alumina, Zirconia, hydroxiapatite, porceins, phosphates, bioactive glass
Composites	Poly(methyl-methacrilate)-glass fibers, Bisphenol-A-glycidyl-methacrylate-quartz/silica fillers

**Table 2:** Degradation of a polymer-based implant over time in 0.154 M sodium chloride [16].

**Material**	**Immersion Time (days)**	**Compressive Modulus (MPa)**	**Compressive Strength (MPa)**	**Fracture Strain (%)**
Poly-lactic acid	0	200 ± 45	14 ± 5	30 ± 9
Poly-lactic acid	3	22 ± 3	5 ± 3	35 ± 8
Poly-lactic acid	14	39 ± 9	4 ± 2	32 ± 2
**Material**	**Immersion Time (days)**	**Compressive Modulus (MPa)**		**Fracture Strain (%)**
Poly-lactic acid	0	200 ± 45	14 ± 5	30 ± 9
Poly-lactic acid	3	22 ± 3	5 ± 3	35 ± 8
Poly-lactic acid	14	39 ± 9	4 ± 2	32 ± 2

**Table 3:** A comparison of the mechanical properties of various metallic implants with natural bone [24].

Property	Natural Bone	Magnesium Alloys	Titanium Alloys	Cobalt–Chromium Alloys	Stainless Steels
Density (g/cm^3^)	1.8–2.1	1.74–2.0	4.4–4.5	8.3–9.2	7.9–8.1
Elastic modulus (GPa)	3–20	41–45	110–117	230	189–205
Yield strength (MPa)	130–180	85–190	758–1117	450–1000	170–310

**Table 4:** A comparison of the composition of the blood plasma with the most commonly used simulated in-vitro electrolytes [56].

Quantity of Ions and Organic Components		Blood Plasma	Hanks’ Solution	Ringer’s Solution	DMEM	PBS	Original SBF	c-SBF	r-SBF	i-SBF	m-SBF
Na^+^	(mmol L^−1^)	142	142	130	1273	157	142	142	142	142	142
K^+^	(mmol L^−1^)	5	5.9	4	5.3	4.1	5	5	5	5	5
Ca^+^	(mmol L^−1^)	2.5	1.3	1.4	1.8	_	2.5	2.5	2.5	1.6	2.5
Mg^2+^	(mmol L^−1^)	1.5	0.8	_	0.8	_	1.5	1.5	1.5	1	1.5
HCO_3_^−^	(mmol L^−1^)	27	4.2	_	44.1	_	4.2	4.2	27	27	10
Cl^−^	(mmol L^−1^)	103	145	109	90.8	140	148.8	147.8	103	103	103
HPO_4_^2−^	(mmol L^−1^)	1	0.8	_	0.9	11.5	1	1	1	1	1
SO_4_^2−^	(mmol L^−1^)	0.5	0.8	_	0.8	_	0	0.5	0.5	0.5	0.5
Buffer	_	_	_	_	HEPES	_	Tris	Tris	HEPES	HEPES	HEPES
Amino acids	(g L^−1^)	_	_	_	1.6	_	_	_	_	_	_
Glucose	(g L^−1^)	0.65–1.1	1	_	4.5	_	_	_	_	_	_
Albumin	(g L^−1^)	30–55	_	_	_	_	_	_	_	_	_
α-globulins	(g L^−1^)	5–10	_	_	_	_	_	_	_	_	_
β-globulins	(g L^−1^)	6–12	_	_	_	_	_	_	_	_	_
γ-globulins	(g L^−1^)	6.6–15	_	_	_	_	_	_	_	_	_
α_1_-lipoproteins	(g L^−1^)	6–12	_	_	_	_	_	_	_	_	_
Fibronogen	(g L^−1^)	1.7–4.3	_	_	_	_	_	_	_	_	_
cholesterol	(g L^−1^)	1.2–2.5	_	_	_	_	_	_	_	_	_
Fatty acids	(g L^−1^)	1.9–4.5	_	_	_	_	_	_	_	_	_
Lactate	(mmol L^−1^)	0.5–2.2	_	_	_	_	_	_	_	_	_
Urea	(mmol L^−1^)	3–7	_	_	_	_	_	_	_	_	_

DMEM, Dulbecco’s Modified Eagle’s Medium; PBS, phosphate-buffered saline; SBF, simulated body fluid; c-SBF, conventional SBF; r-SBF, revised SBF; i-SBF, ionized SBF; m-SBF, modified SBF.
